# The Aorta Is One Organ; Our Response Is Not: A Global Case for Systems Redesign in Acute Aortic Syndromes

**DOI:** 10.3390/jcm15166363

**Published:** 2026-08-18

**Authors:** Farhin Holia, Aung Ye Oo, Hans-Joachim Schäfers

**Affiliations:** 1 Department of Cardiothoracic Surgery, St Bartholomew’s Hospital, Barts Health NHS Trust, London EC1A 7BE, UK; farhin.holia@nhs.net; 2 William Harvey Research Institute, Queen Mary University of London, London E1 4NS, UK; 3 Department of Thoracic and Cardiovascular Surgery, Saarland University Medical Centre, 66424 Homburg, Saar, Germany; 4 Hospital Universitario Quironsalud, 28223 Madrid, Spain; 5 Department of Thoracic and Cardiovascular Surgery, Westpfalz-Klinikum, 67655 Kaiserslautern, Germany

**Keywords:** acute aortic syndrome, aortic dissection, thoracic aortic aneurysm, global burden of disease, clinical registries, screening, multidisciplinary care pathway, health systems, quality of care, survivorship

## Abstract

The aorta is a single arterial organ, yet the clinical response to its diseases is fragmented across specialties, institutions, and nations. Acute aortic syndromes remain lethal at every stage of the care continuum: a large share of patients with type A dissection die before reaching any hospital, roughly half die before reaching a specialist centre, a substantial proportion are misdiagnosed at first medical contact, and untreated mortality has historically been estimated at approximately one to two percent per hour. Reported 30-day mortality differs across surgical registry cohorts—for example, 7.6% in the Japan Cardiovascular Surgery Database and 16.9% in the cited GERAADA cohort—but such ecological comparisons are descriptive and cannot identify the contribution of organisational, biological, clinical, or ascertainment differences. This review synthesises registry, population, and health-services data from Europe, Japan, North America, and low- and middle-income settings to characterise where the pathway fails and what each system has already demonstrated. We argue that the next material advance in aortic care will come not from any single innovation in isolation but from the architecture that connects prevention, diagnosis, treatment, and follow-up and that system redesign and technological progress are complementary rather than competing; we propose a five-pillar framework (Prevention, Presentation, Pathway, Person, and Learning) mapped throughout to published exemplars and future research priorities.

## 1. Introduction

The aorta is not a passive conduit. It is the largest arterial organ in the body, a layered and living structure whose intima, media, and adventitia together integrate haemodynamic load, endothelial biology, and matrix turnover across the whole of a life. Its diseases are, correspondingly, diseases of the whole organ rather than of an isolated segment. A tear at the root propagates to the arch; an aneurysm of the descending thoracic aorta shares genetic and haemodynamic determinants with abdominal aortic disease [1]; and a bicuspid aortic valve with an ascending dilatation represents two expressions of a single developmental biology [2]. The majority of thoracic aortic aneurysms remain clinically silent until dissection or rupture supervenes. The organ is silent until, abruptly, it is not.

The clinical response to this single organ, by contrast, is profoundly fragmented. Cardiology owns the valve; cardiac surgery owns the root and ascending aorta; vascular surgery owns the descending and abdominal segments; interventional radiology owns much of the endovascular repertoire; emergency medicine owns the acute presentation; primary care owns the long-term follow-up; and clinical genetics owns the family. Each of these disciplines has matured impressively in isolation. The disease, however, has not obligingly fragmented along the same lines. Acute aortic dissection begins years before the tear, in a family history never elicited and an aorta never measured, and its outcome is frequently decided long before, and long after, the operation itself. We continue to manage a single organ as a set of anatomical territories and a lifelong disease as a discrete episode. This review is concerned with the architecture that closes that gap.

Our central contention is that potentially modifiable organisational factors plausibly contribute to aortic outcomes alongside disease severity, clinical presentation, treatment strategy, and access to care. Cross-system registry differences cannot independently estimate that contribution because datasets differ in case ascertainment, denominator definition, referral patterns, operability, case mix, endpoints, and reporting practice. They can, however, identify recurrent care-process failures, generate hypotheses, and point to organisational interventions that warrant prospective evaluation. This is a narrative review; it does not present new patient-level data. We searched the peer-reviewed literature and major clinical registries for evidence on the epidemiology, care pathways, and outcomes of acute aortic syndromes and thoracic aortic disease, with an emphasis on population-based and registry sources that capture pre-hospital and system-level attrition. The literature search drew on MEDLINE/PubMed and Embase from database inception to 2025, combining terms for aortic dissection, acute aortic syndrome, thoracic and abdominal aortic aneurysm, and screening with terms for registries, care pathways, networks, mortality, and health systems, supplemented by hand searching of reference lists and relevant society guidelines. National exemplars were selected on the basis of mature national or regional systems reporting published, quantitative outcome or process data that illustrate a distinct and transferable organisational lesson, rather than through a formal systematic protocol; this remains a narrative synthesis and is not intended as a systematic review. We organise the evidence around the continuum of care and conclude with a structured, exemplar-anchored framework and a set of prioritised research questions.

Systems approaches to acute aortic syndromes have been described previously, including multidisciplinary high-volume care and regional management flows [3]. The present review therefore positions its five-pillar framework as an evidence-informed synthesis and implementation proposal, rather than as the first systems account of acute aortic disease.

## 2. Where the Pathway Fails: Three Points of System Failure

It is useful to locate failure at three distinct points along the care continuum: before the tear, between the onset of pain and the operation, and after surgical repair. At each point, the deficit is one of coordination and uptake rather than of fundamental knowledge.

### 2.1. Before the Tear: An Unclaimed Reservoir of Preventable Risk

Acute aortic dissection is more inherited than commonly appreciated. First-degree relatives of index patients carry a substantially elevated risk of both aneurysm and dissection, and heritability estimates for thoracic aortic disease are high [2,4]. In first-degree relatives of individuals with a bicuspid aortic valve, meta-analysis demonstrates that a meaningful minority themselves carry a bicuspid valve or aortic dilatation, establishing a clear yield for structured family screening [2]. An expert consensus statement on cardiogenetic evaluation and family screening for thoracic aortic disease was published in 2018 [5]. What is missing, therefore, is not only knowledge of who to screen but reliable systems that identify, contact, and retain at-risk relatives.

A second reservoir sits in plain sight within imaging already performed for other reasons. In targeted lung-health-check cohorts, thoracic aortic dilatation can be detected incidentally on low-dose CT and referred for confirmatory assessment, although current evidence is regional and largely observational [6,7]. A single referral-centre retrospective series found that aneurysms identified after 2013 were modestly smaller at presentation than those identified earlier; this temporal association does not establish that lung-cancer screening caused earlier detection [8]. Every uncalled relative and every unmeasured aorta represent a potential opportunity for earlier risk assessment, but the yield, clinical consequences, and cost-effectiveness of systematic pathways require prospective evaluation.

### 2.2. Between Pain and Operation: Information That Dies in Transit

Once dissection occurs, time becomes the dominant variable. In untreated type A dissection, the mortality hazard has historically been estimated at roughly one to two percent per hour during the first 24 to 48 h; this is a historical rule of thumb derived from early natural-history series [9,10] and should be interpreted as an approximate historical estimate rather than a contemporary population parameter. National English reporting has estimated that around one-fifth of patients with acute aortic dissection die before reaching any hospital, and around half die before reaching a specialist centre [11]. Misdiagnosis compounds delay. A systematic review found that a substantial proportion of dissections are misdiagnosed at first contact, and a retrospective observational study associated missed diagnosis with an initially ischaemic electrocardiogram and an initial working diagnosis of myocardial infarction [12,13].

The characteristic failure mode in this interval is sequential loss of information across professional handovers. A radiologist may identify the dissection, while the surgeon does not yet know of it; the surgeon may accept the patient, while the receiving theatre and anaesthetic team are not yet prepared. These are not, for the most part, failures of individual competence. They are the predictable consequence of a pathway in which no one has been made responsible for the boundary between one professional and the next.

Formal regional protocols have previously aimed to shorten the time from hospital arrival to diagnosis and treatment through standardised multidisciplinary coordination [14]. In a retrospective before-and-after study of acute aortic disease, implementation of a critical pathway was associated with shorter door-to-CT and time-to-intervention intervals; its observational design and mixed disease population limit causal interpretation [15].

### 2.3. After the Operation: The Person Left Behind

A technically successful operation does not guarantee a successful outcome for the patient, and operative survival is only the first of several thresholds. In a selected cohort of survivors of type A repair with residual aortic dissection and available follow-up imaging, 46.1% experienced a dissection-related event over a mean 20.4 months; this figure applies to that survivor subgroup rather than to all patients undergoing type A repair [16]. Surveillance after acute dissection is also an implementation challenge: in a single-centre cohort of 272 acute-dissection survivors, including both type A and type B disease, only 9.6% were fully concordant with recommended imaging in the first year [17]. Separately, qualitative work documents post-traumatic stress, chronic pain, persistent anxiety about recurrence, and other survivorship needs among people with or at risk for aortic dissection [18]. These are linked care-continuity problems, but their frequency and determinants require diagnosis- and procedure-specific measurement.

**Figure 1:**
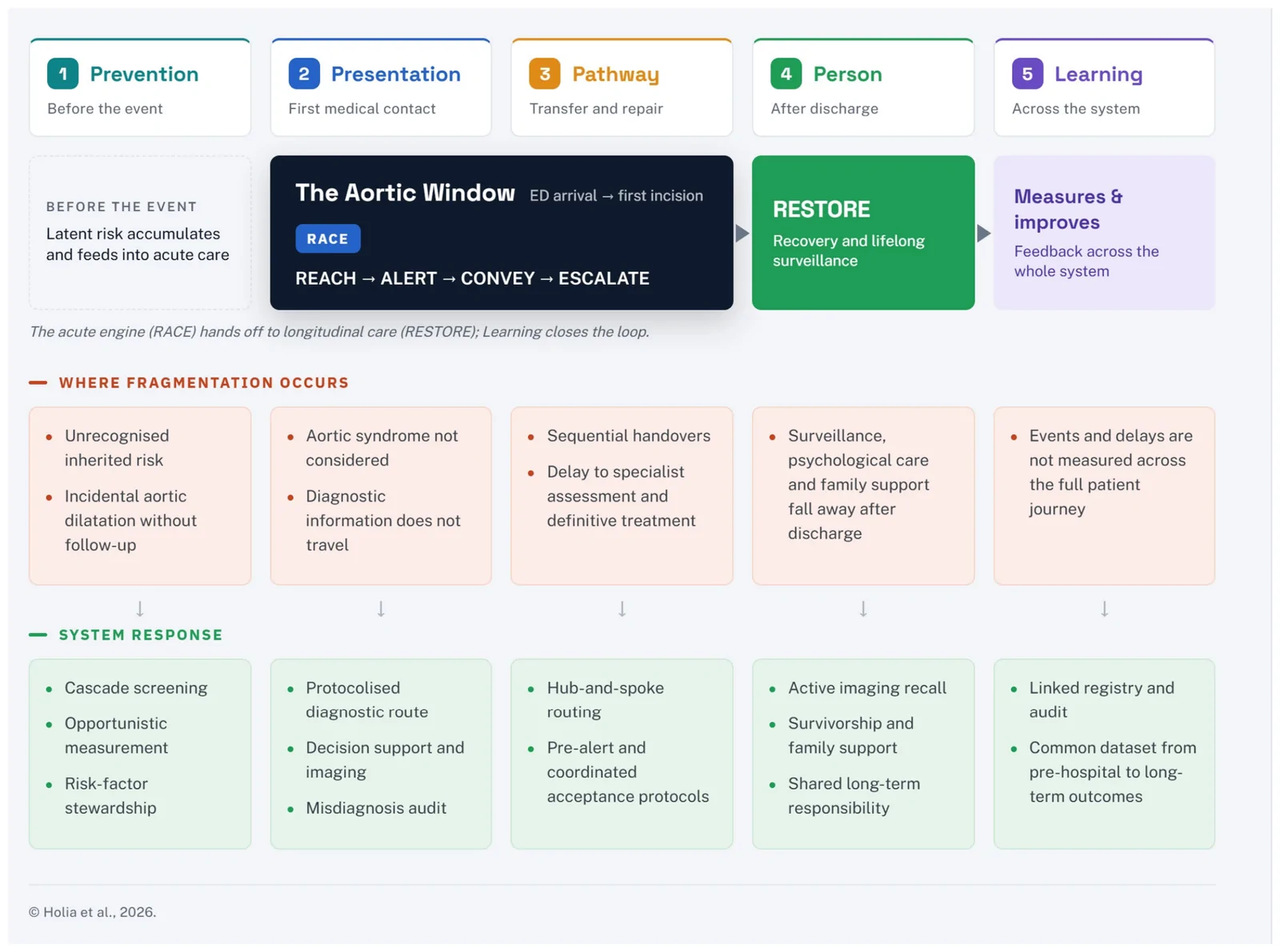
A five-pillar framework for integrated acute aortic-syndrome care. The five pillars are Prevention, Presentation, Pathway, Person, and Learning. The previously proposed Aortic Window framework operationalises the acute interval from emergency-department arrival to first incision through RACE (Reach → Alert → Convey → Escalate); RESTORE denotes recovery and lifelong surveillance [19]. The figure is conceptual, not a quantitative cascade or comparison of outcome estimates. Source-specific outcome estimates and their metadata are reported in Table 1.

Figure 1 sets out these three points of failure alongside the corresponding system responses, which are developed as a five-pillar framework in Section 5.

**Table 1:** Reported outcome estimates in acute type A aortic dissection. For every estimate, the study period, denominator, exact endpoint, and adjustment/uncertainty status are stated. Estimates differ in endpoint, denominator, study period, case mix, and adjustment; they are presented as source-specific values rather than on a common comparative axis. Volume effects are within-study contrasts, not cross-national rankings.

Study/Cohort	Period/Denominator	Exact Endpoint	Reported Estimate	Adjustment/Uncertainty	Source
National/registry cohorts—source-specific values; not mutually comparable					
Japan, JCVSD	2008–2015; n = 11,843 surgical patients	30-day mortality; operative mortality reported separately	7.6% (30-day); 9.5% (operative)	Displayed rates unadjusted; study modelled risk-adjusted operative-mortality trends; CI for overall crude rates not reported in abstract	Abe et al. [20]
Germany, GERAADA	2006–2010; n = 2137 surgically treated patients	30-day mortality	16.9%	Registry cohort; CI for crude rate not reported in registry overview	Boening et al. [21]
Regional network—within-system before/after comparison; concurrent clinical changes					
Udine, before network	Before 2016; n = 90; exact calendar range not reported in retrieved paper text	Early mortality	13%	Unadjusted before–after comparison; *p* = 0.013 for between-group mortality; CI for crude rate not reported in retrieved paper text	Vendramin et al. [22]
Udine, after network	2016 onward; n = 109; exact calendar range not reported in retrieved paper text	Early mortality	4%	Unadjusted before–after comparison; concurrent differences in arch replacement, cannulation, and circulatory-arrest time; CI for crude rate not reported in retrieved paper text	Vendramin et al. [22]
Volume effects—within-study contrasts only; not cross-national performance rankings					
UK surgeon annual volume <4 vs. ≥4	2007–2013; 1550 operations; 249 surgeons	In-hospital mortality	19.3% vs. 12.6%	Unadjusted stratum comparison (*p* = 0.015); separate risk-adjusted volume analysis; CI for crude rates not reported in abstract	Bashir et al. [23]
US low- vs. high-volume centres	2005–2014; NIS estimate n = 25,231; low n = 10,115; high n = 6920	In-hospital mortality	21.5% vs. 11.6%	Unadjusted stratum comparison; adjusted low- vs. high-volume OR 2.10 (*p* < 0.001); CI not reported in abstract	Dobaria et al. [24]

Notes: “Unadjusted” refers to the displayed crude rate; adjusted associations, where available, are reported separately. Each estimate row identifies the adjustment status and states whether a confidence interval for the displayed crude rate was reported in the source record available for this review, rather than implying one. Endpoints are not interchangeable: 30-day, operative, early, and in-hospital mortality use different time windows and denominators.

## 3. What the Contemporary Literature Has Not Yet Solved

Even within the best-resourced systems, four questions remain genuinely open, and each is central to any credible redesign because each defines a boundary of current practice.

First, we currently image on a fixed schedule that the underlying biology does not respect. A 2025 systematic review and meta-analysis of thoracic aortic aneurysm growth rates reported slow mean growth across syndromic, bicuspid, and sporadic disease and concluded that no established predictor of growth exists and that annual surveillance is likely too frequent for many patients [25]. The right surveillance interval for the individual patient is not knowable at present.

Second, the management of chronic type B dissection is marked by persistent transatlantic disagreement. A 2025 critical review of guidelines documented inconsistent thresholds for pre-emptive thoracic endovascular repair and divergent recommendations on surveillance modality and interval [26] against a background of poor real-world surveillance adherence [27] that a scientific statement on chronic dissection imaging has also highlighted [28].

Third, the biology of aortic instability, that is, the transition from a stable dilatation to catastrophic dissection, remains incompletely understood, as recent mechanistic work makes explicit [29,30]. Maximal diameter persists as the dominant operative threshold not because it is demonstrably the correct discriminator of risk but because it is very nearly the only quantitative discriminator currently available at the bedside. A prospective histopathology study of surgical aortic samples reported differences in medial and myxomatous changes between aneurysm and dissection samples and found that moderate or severe medial degeneration was associated with dissection and that its histopathological score predicted in-hospital and overall mortality [31]. These findings support further study of tissue-level features as potential biomarkers but do not yet establish a bedside replacement for diameter.

Fourth, the lived experience of patients and at-risk relatives, comprising the informational, psychological, and continuity-of-care burdens they carry, has been characterised qualitatively but is largely unmeasured by the systems that treat them [18]. This is itself an evidence gap: the field lacks validated, aorta-specific patient-reported outcome measures and, correspondingly, a systematic accounting of survivorship as a system outcome. None of these four problems can be solved by a single specialty, or a single country, acting alone.

## 4. The Global Ecosystem: What Each System Teaches

Cross-system variation in aortic outcomes is clinically important but not causal evidence in itself. Each registry or network captures a different population, denominator, care setting, and endpoint. Read together, these sources identify care-process questions and possible organisational exemplars; they do not provide a common performance ranking. Table 2 collates the distinctive contribution of each ecosystem and the transferable lesson that may be tested or adapted.

The International Registry of Acute Aortic Dissection (IRAD) provides longitudinal, cross-institutional observational data across North America, Europe, and Japan [32]. Germany’s GERAADA registry provides a structured, subspecialty dataset; in its validated 2006–2010 surgical cohort, 30-day mortality after type A repair was 16.9%, and the pre-operative risk score has subsequently been evaluated in Japanese cohorts [21,33,34]. These registry results are defined benchmarks, not direct comparators for differently ascertained cohorts.

Japan’s JCVSD provides another surgical benchmark: among 11,843 operations undertaken from 2008 to 2015, 30-day mortality was 7.6%, and operative mortality was 9.5% [20]. Differences between JCVSD, GERAADA, and other registries must be interpreted with attention to ascertainment, denominator definition, case mix, and endpoint. Population-based Japanese data that incorporate patients dead on arrival and post-mortem imaging show how hospital-based series can underestimate burden [35].

The Nordic countries provide relevant screening evidence for abdominal aortic aneurysm, although this should not be extrapolated directly to thoracic aortic disease. Sweden’s national programme of ultrasound screening for abdominal aortic aneurysm in 65-year-old men, compared against demographically and structurally similar but unscreened Finland, is associated with a decline in ruptured-aneurysm repair and improved outcomes [38], while a Danish randomised screening trial reported a reduction in aneurysm-related mortality and emergency operations over ten years [39]. Neither result establishes a benefit for thoracic-aorta screening. Network studies in France and Italy offer organisational exemplars, but their designs do not isolate the effect of network implementation from concurrent clinical change. In the Udine before–after cohort, early mortality fell from 13% to 4%, diagnosis-to-surgery time fell from 210 to 160 min, and 5-year survival changed from 70% to 87%; the post-network group also had more arch replacement, more frequent axillary cannulation, and shorter circulatory-arrest times [22]. These findings support prospective evaluation of integrated network models rather than a causal attribution to any single component.

**Figure 2:**
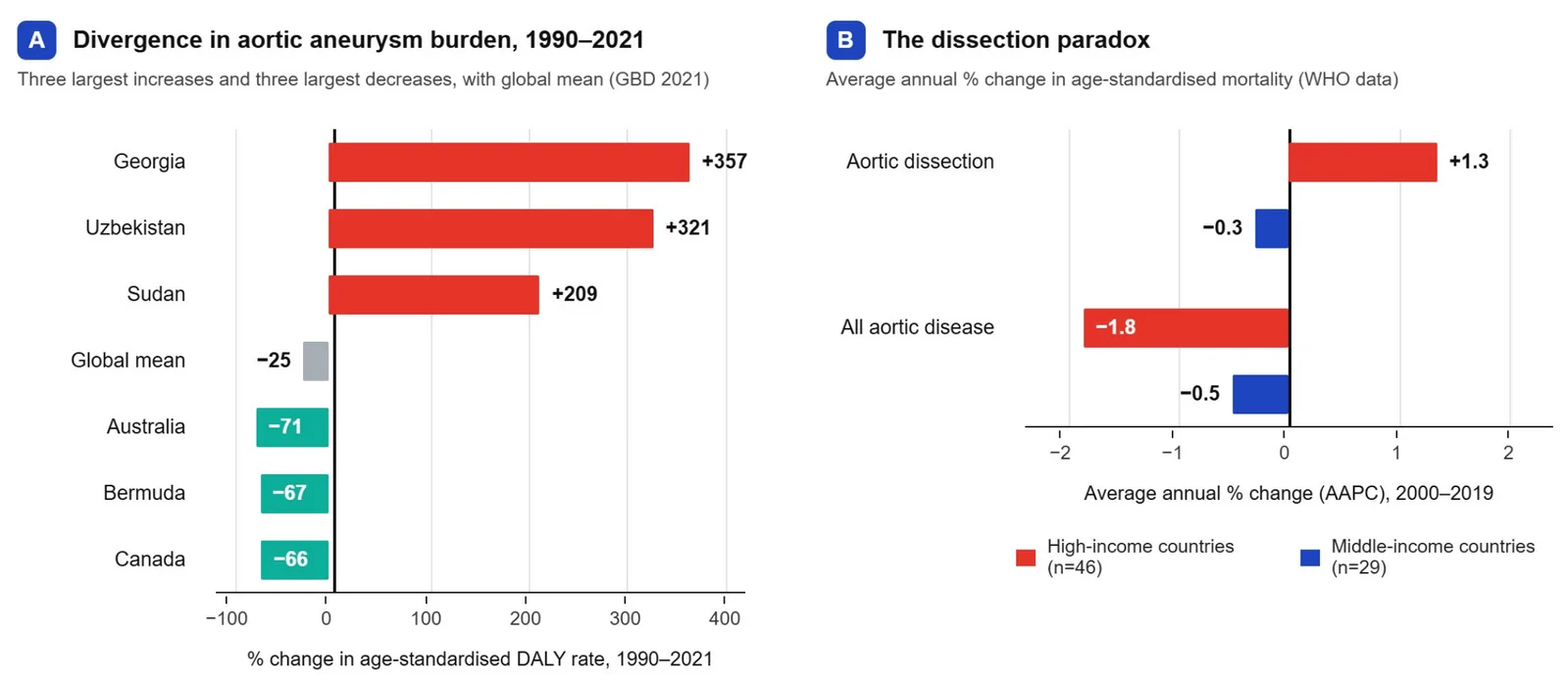
(**A**): Percent change in the age-standardised aortic aneurysm DALY rate between 1990 and 2021 for the three countries with the largest increases and the three with the largest decreases in Global Burden of Disease 2021 estimates, alongside the global-mean change [45]. The global age-standardised aneurysm death and DALY rates fell over the period, a decline driven predominantly by high-income countries. (**B**): the dissection paradox. Across 2000–2019 WHO mortality data, age-standardised mortality from all aortic disease declined in both middle- and high-income countries, yet age-standardised mortality from aortic dissection specifically rose in high-income countries even as aneurysm mortality fell [46]. Values are drawn from the sources cited in the text.

English national data have also described decade-long trends in surgery for acute type A dissection [47]. The United States illustrates both the volume–outcome relationship and the price of ignoring equity: high-volume centres achieve lower mortality and costs [24,41], yet lower socioeconomic status is independently associated with higher in-hospital mortality after type A repair [42,43]. Volume without equity is not a solved system. Finally, low- and middle-income countries reveal what happens when disease burden outpaces infrastructure. Global aortic morbidity and mortality rose substantially between 1990 and 2019, with the steepest increases in lower-income settings [44], and Global Burden of Disease estimates record the largest age-standardised aneurysm DALY increases in countries such as Georgia, Uzbekistan, and Sudan, against decreases in high-income settings (Figure 2A) [45]. Across 2000–2019 WHO mortality data, age-standardised mortality from all aortic disease fell by 1.8% per year in high-income countries, yet age-standardised mortality from aortic dissection specifically rose by 1.3% per year over the same period (Figure 2B) [46]. The paradox is diagnostic: the components of aortic care that have improved most in high-income systems, namely elective aneurysm repair, hypertension control, and imaging access, are not the components that decide dissection outcomes. No system, however well-resourced, is converging on a solved endpoint.

## 5. A Framework for Systems Redesign: Five Pillars

We propose that the evidence above be operationalised through five linked pillars: Prevention, Presentation, Pathway, Person, and Learning. These are not aspirations; each is an operational commitment mapped to an exemplar the world has already produced (Table 3). To keep the evidential status of each commitment explicit, we annotate the pillars below as resting on evidence-supported intervention (E), expert recommendation (R), or research priority (P), recognising that many combine more than one. The framework is deliberately disciplined: it relies on existing imaging, existing emergency infrastructure, existing clinics, and existing audit machinery and uses the ecosystem lens to indicate which existing resource each system should build on next.

Pillar 1, Prevention, comprises (E/R) a structured familial cascade for every index case of bicuspid valve, heritable thoracic aortic disease, and early dissection [2,48]; opportunistic, increasingly AI-assisted aortic measurement on chest CT performed for any indication [49,50]; an aortic pathway embedded within every established lung-cancer-screening programme [6,7]; a nationally organised abdominal aneurysm screening programme on the Swedish and English models [38,51]; and disciplined blood-pressure stewardship in confirmed aortopathy, given the strong association between uncontrolled hypertension and dissection risk in population-based data [9]. Whether specific blood-pressure targets in confirmed aortopathy reduce dissection incidence at scale is itself a research priority (Section 8).

Pillar 2, Presentation, requires (R) a protocolised diagnostic pathway for acute chest, back, and abdominal pain in which dissection is actively excluded rather than incidentally considered, particularly in patients with ischaemic ECG changes in whom it is systematically under-recognised [12,13], supported by emergency-department decision support and by formal audit of misdiagnosis and 14-day repeat presentations.

For acute type A dissection, the recently proposed “aortic window” offers an operational model: a named RACE/RESTORE activation pathway and emergency-department-to-incision time as a whole-system process metric. Its targets are explicitly prospective and require validation before being adopted as standards [19].

Pillar 3, Pathway, defines (E/R) an ambulance-to-aortic-centre route with pre-alert to the receiving surgeon, anaesthetist, and theatre; centralised acceptance protocols that eliminate sequential information loss between radiologist, surgeon, accepting centre, and anaesthetist; and hub-and-spoke consolidation of type A repair in high-volume centres on the French and Italian organisational exemplars [22,40]. The network and volume evidence is observational, so pre-alert, acceptance protocols, and routing remain recommendations for local adaptation rather than universally validated standards.

A direct-to-operating-room transfer program provides a further operational exemplar: in a single-centre retrospective pre/post cohort, it was associated with shorter transfer-acceptance-to-operating-room and arrival-to-operating-room intervals. The observed mortality signal was hypothesis-generating rather than causal proof [52].

Pillar 4, Person, mandates (R/P) structured post-operative surveillance imaging with active recall of non-attenders. In a mixed acute-dissection survivor cohort, full first-year imaging concordance was low, supporting surveillance as a care-continuity target while not establishing the rate for every diagnosis or procedure [17]. The pillar also proposes systematic screening for post-traumatic stress, chronic pain, and psychological morbidity and a formal patient-and-family voice drawing on qualitative survivorship evidence [18]. The distal-event rate after type A repair is reported separately for the selected residual-dissection survivor cohort described above [16].

Sex is a cross-cutting determinant that runs through several of these pillars and warrants explicit attention. Although some aortic diseases are less frequent in women, women may present later and less typically, be referred and investigated for dissection less readily, and, in several thoracic and abdominal aortic settings, undergo intervention less frequently and experience worse outcomes; anatomical differences also affect suitability for endovascular repair and appropriate surveillance. A systems response should therefore account for sex in diagnosis, referral, anatomical assessment, treatment selection, and follow-up rather than treating it as incidental [53,54].

Pillar 5, Learning, establishes (R) a national acute-aortic-syndrome registry on the GERAADA and IRAD template, capturing pre-hospital, emergency-department, transfer, operative, and long-term outcomes [21,32]; formal audit of pre-specialist mortality, misdiagnosis, weekend effects, and surveillance concordance; a cross-national data-harmonisation channel modelled on established joint registry initiatives [55]; and medico-legal case review used as a system-improvement input rather than solely as an instrument of individual accountability.

The Collaborative Acute Aortic Syndrome Project protocol provides a complementary model for pathway measurement, designed to relate timing, clinical and demographic factors, and outcomes across multiple centres. As a protocol, it should be cited as an example of an active measurement agenda rather than completed intervention evidence [56].

**Table 3:** The five-pillar framework for aortic systems redesign, with the operational commitment, existing infrastructure it builds on, published evidence anchor, and country exemplar for each pillar.

Country Exemplar	Evidence Anchor	Existing Infrastructure It Builds On	Operational Commitment	Pillar
Sweden, England (NAAASP), Denmark, Netherlands (BAV cascade)	Family screening yield in BAV [2]; heritable-aortopathy consensus [4,48]; incidental aortic dilatation in lung-cancer screening [6,7,8]; Sweden vs. Finland AAA outcomes [38]; Viborg RCT [39]; NHS AAA screening impact [51]; hypertension–dissection association [9]	Cardiogenetics clinics; radiology PACS; national lung-cancer-screening programmes; existing AAA screening bodies; primary care	Familial cascade screening; opportunistic aortic measurement on every chest CT; aortic pathway embedded in lung-cancer screening; national AAA screening; BP stewardship in confirmed aortopathy	1. Prevention (E/R)
UK ED audit, Nordic diagnostic-pathway studies	Misdiagnosis systematic review [12]; missed-dissection risk factors [13]	Emergency departments; existing chest-pain pathways; radiology CT infrastructure	Protocolised diagnostic pathway that actively excludes dissection in acute chest/back/abdominal pain; ED decision support; audit of misdiagnosis and 14-day representation	2. Presentation (R)
France (Paris), Italy (Udine)	Paris regional network [40]; Udine hub-and-spoke before-and-after [22]	Ambulance services; existing aortic centres; regional cardiac networks	Ambulance-to-aortic-centre routing with pre-alert; centralised acceptance protocol eliminating sequential handover loss; hub-and-spoke consolidation of type A repair	3. Pathway (E/R)
Emerging survivorship programmes in North America and Europe	Distal-event rate after type A repair [16]; qualitative survivorship evidence [18]	Existing follow-up clinics; recall registries; imaging bookings	Structured post-operative surveillance imaging with active recall; systematic screening for PTSD, chronic pain, psychological morbidity; formal patient-and-family voice	4. Person (R/P)
Germany (GERAADA), Japan (JCVSD), UK (NACSA), international (IRAD)	GERAADA structure [21]; IRAD 25-year synthesis [32]; STS/JCVSD data harmonisation [55]	Existing national audit bodies (NACSA, GERAADA); IRAD; STS/JCVSD data-sharing precedents	National acute-aortic-syndrome registry (pre-hospital → long-term); audit of pre-specialist mortality, misdiagnosis, weekend effect, surveillance concordance; cross-national data harmonisation; medico-legal case review as system input	5. Learning (R)

## 6. Governance: National First, Then Global

Implementation requires governance at two levels. Nationally, a joint aortic-systems standing committee, convened under existing cardiac, vascular, and cardiology societies with defined representation from radiology, emergency medicine, primary care, critical care, clinical genetics, patients, and policymakers, would set minimum standards for each pillar and maintain an outcome-reporting dataset comparable to NACSA and GERAADA [21,36]. Globally, a networked structure linking IRAD, GERAADA, JCVSD, Swedvasc, NACSA/NAAASP, and emerging data collaborations in lower-income settings could maintain a common minimum dataset and an explicit capacity-building programme, given a burden trajectory that high-income infrastructure alone cannot absorb [44,45,46]. The paramedic and emergency physician stand at the start of the chain; the radiologist and surgeon stand at its centre; the intensivist and the family stand at its end; the policymaker and the patient stand throughout. Each of these actors holds a link. A pathway fails where no one has been made responsible for what happens between two of them.

## 7. Scope and Boundaries: What This Framework Does Not Advocate

It is important to state clearly what this argument does not entail. It is neither a call for population-wide CT screening of the thoracic aorta, as radiation exposure is a material harm, and yield, benefit, and cost-effectiveness would need prospective demonstration before such an approach could be adopted [57], nor a call for the over-medicalisation of small aortic dilatations, which risks anxiety, over-investigation, and unnecessary intervention. The framework does not require a single-payer environment: its commitments can be adapted at the level of a regional network, a hospital system, or a professional society.

## 8. Discussion and Future Directions

The evidence assembled here supports a deliberately limited organising claim: potentially modifiable features of care delivery may influence aortic outcomes, but their independent contribution cannot be estimated from ecological comparisons of registries or systems. The approximately fivefold spread among selected reported crude mortality estimates is descriptive, not a causal effect estimate. More informative evidence comes from studies that evaluate a defined pathway, transfer process, volume exposure, or network intervention within a specified population and endpoint; even these observational studies require careful attention to patient selection, concurrent operative and perioperative changes, and residual confounding. The corresponding research agenda follows directly from the four unsolved problems identified in Section 3 and from the structure of the five pillars.

Technological progress should be evaluated as part of, rather than in competition with, system redesign. For selected acute arch and descending-aortic presentations, a durable proximal seal in zone 2 may require preservation of left-subclavian flow. The modular retrograde-branch Gore TAG Thoracic Branch Endoprosthesis (TBE) and the integrated unibody Castor and second-generation Cratos platforms are dedicated single-branch options for this purpose. Their regulatory status, availability, branch geometry, access requirements, and landing-zone requirements differ by platform and jurisdiction; the term ‘off-the-shelf’ should therefore not be applied uniformly to all three. In anatomically suitable patients, these technologies can support a totally endovascular zone-2 strategy and may avoid surgical left-subclavian debranching [58]. Evidence of procedural advantage remains observational. In a single-centre retrospective comparison, branched endografting was associated with shorter operative time and hospital stay than TEVAR with surgical left-subclavian revascularisation, but the study was non-randomised and cannot establish comparative benefit [59]. In early selected series, TBE achieved maintained left-subclavian patency and no 30-day major complications in 20 acute-pathology patients; a subsequent seven-centre retrospective acute cohort reported 107 urgent/emergent cases, 99% technical success, and 2% 30-day mortality [60,61]. A small Western Castor series likewise reported preserved branch patency and no 30-day deaths but included 15 elective patients and requires longer follow-up [62]. These reports support feasibility, not comparative effectiveness or a system-level outcome claim.

### Five Priorities Stand Out

First, the field needs biomarkers and imaging-derived metrics of aortic instability that improve upon maximal diameter as a discriminator of dissection risk, so that surveillance intervals can be matched to individual biology rather than to a fixed calendar [25,29,30]. Second, the benefit of abdominal aneurysm screening should be formally evaluated for the thoracic aorta, including opportunistic, AI-assisted measurement on already-acquired CT, with prospective assessment of yield, cost-effectiveness, and harm [8,49,50]. Third, the associations observed after regional-network implementation in Udine and Paris should be tested prospectively and at scale in other health systems, with pre-specified process metrics such as diagnosis-to-surgery interval and pre-specialist mortality; such studies must measure concurrent changes in operative strategy, cannulation, circulatory-arrest management, and perioperative care [22,40]. Fourth, survivorship, measured through surveillance concordance, psychological morbidity, and the patient and family experience, should be assessed routinely as an outcome of the system rather than left to qualitative study alone [17,18]; a prerequisite is the development and validation of aorta-specific patient-reported outcome measures. Fifth, whether protocolised blood-pressure stewardship in confirmed aortopathy actually reduces dissection incidence at population scale is not established and warrants a pragmatic trial.

This review has limitations. It is a narrative rather than a systematic synthesis, and the cross-system comparisons it draws are necessarily ecological: registries differ in case ascertainment, denominator definition, patient selection, referral patterns, operability, case mix, endpoints, treatment strategy, operative expertise, perioperative management, and reporting practice. Their outcome differences therefore cannot quantify, or be attributed independently to, organisation. Population-based datasets that capture pre-hospital death, such as the Oxford Vascular Study and the Japanese autopsy-imaging cohorts [11,35], consistently report higher case fatality than hospital-based registries, which underlines how much of the true burden remains invisible to conventional surgical series and how cautiously headline mortality figures should be compared. Centralisation can also add transfer distance and time; any hub-and-spoke model needs pre-defined triage, acceptance, transport, and escalation protocols. Family-screening cascades and active surveillance recall require workforce, imaging capacity, data systems, and sustainable funding. Full implementation may be infeasible in resource-constrained settings, so the framework is intended to be modular: systems can prioritise a locally feasible component and evaluate its process and patient outcomes before extension.

## 9. Conclusions

The aorta is a single organ, and its diseases are diseases of the whole organ and of the whole life. The evidence from a dozen national systems shows that the response to those diseases is fragmented, that the fragmentation is costly, and that the cost is not fixed. When a system fails, an individual clinician is too often named; when a system is fixed, no one is. The aortic community, spanning surgical, medical, imaging, emergency, primary-care, critical-care, genetic, patient, and policy domains across every income setting, is well placed to ensure that it is the second sentence, and not the first, that is written next. The next material advance in aortic care will come not from any single device, scan, or guideline in isolation but from the system that ensures such advances reach the right patient in time; system redesign and technological innovation are complementary, and progress will depend on both. **The aorta is one organ. Our response is not—yet**.

## Figures and Tables

**Table 2:** Ecosystem exemplars: the distinctive achievement of each system and the transferable lesson it offers.

System	Instrument	Distinctive Achievement	Transferable Lesson	Reference
International (IRAD)	Voluntary multicentre registry	25 years of harmonised observational data across three continents	Longitudinal, cross-institutional data are a prerequisite for improvement	[32]
Germany (GERAADA)	Prospective all-comers type A registry	Validated 30-day mortality benchmark (16.9%) and externally validated risk score	A defined registry benchmark and common risk language for a heterogeneous emergency	[21,33,34]
Japan (JCVSD)	National cardiovascular surgical database	30-day mortality 7.6% across 11,843 type A operations	A large surgical registry provides a benchmark, not a direct cross-system performance comparison	[20]
Japan (population data)	Autopsy-imaging denominator correction	True dissection incidence roughly double previous hospital-based estimates	Only population-level ascertainment reveals the real burden	[35]
United Kingdom (NACSA)	National mandatory audit	Surgeon volume identified as stronger determinant of mortality than centre volume; unwarranted regional variation named	An audit system willing to name its own variation is itself an improvement instrument	[23,36,37]
Sweden/Finland (Swedvasc)	National AAA screening compared with unscreened neighbour	Reduced ruptured-aneurysm repair and improved outcomes attributable to screening	Structured population screening changes the population outcome	[38]
Denmark (Viborg RCT)	Randomised AAA screening trial	Substantial reduction in aneurysm-related mortality and emergency operations at 10 years	Screening effects are durable when the programme is durable	[39]
France (Paris network)	Regional dedicated aortic network	Survival improved after implementation of a regional dedicated aortic network	A regional network is an organisational exemplar requiring context-specific evaluation	[40]
Italy (Udine hub-and-spoke)	Before-and-after regional network	Before–after cohort: early mortality 13% → 4%; diagnosis-to-surgery 210 → 160 min; 5-year survival 70% → 87%, alongside concurrent operative changes	An integrated network is a testable organisational model; the before–after design cannot isolate any one component	[22]
United States (volume + equity)	Multicentre observational data	High-volume mortality/cost advantage coexists with socioeconomic mortality gradient	Volume without equity is not a solved system	[24,41,42,43]
LMIC/GBD	Global disease-burden modelling	Burden rising fastest where infrastructure is thinnest; dissection mortality rising in HICs	The unfinished part of the global problem is where the infrastructure is not	[44,45,46]

## Data Availability

No new data were created or analysed in this study. Data sharing is not applicable to this article.
